# Translational cancer research – a coherent cancer research continuum

**DOI:** 10.1002/1878-0261.12450

**Published:** 2019-02-01

**Authors:** Ulrik Ringborg

**Affiliations:** ^1^ Cancer Center Karolinska Karolinska University Hospital Solna Stockholm Sweden

**Keywords:** prevention, the cancer research continuum, therapeutics, translational cancer research

## Abstract

The main components of the cancer research continuum are basic/preclinical research, early and late clinical research and, after the adoption of an innovation by the healthcare or health organisations, outcomes research. Translational cancer research, defined as a coherent cancer research continuum, is mandatory to address the increasing burden of cancer effectively. The growing cancer problem can only be significantly modified by concerted action involving prevention to decrease incidence, early detection and treatment to increase the cure rate, and personalised/precision cancer medicine to adapt early detection and treatment to the biology of a tumour with the aim of increasing the cure rate, prolonging survival and improving health‐related quality of life. By definition, translational cancer research for therapeutics has a focus on patients’ needs and for prevention for individuals at‐risk. Consequently, to increase the effectiveness of translational research, the different components of the cancer research continuum need to be better connected to the fundamental aim of a mission‐oriented approach to cancer (Celis and Pavalkis, 2017).

**Abbreviation**
CCC: Comprehensive Cancer Centres

## 1 The cancer research continuum for therapeutics

A critical problem in therapeutics is the suboptimal linkage between different components of the cancer research continuum. Initially, two main gaps were identified, representing early and late translational research (McGartland Rubio *et al*., 2010; Celis and Pavalkis, 2017; see also http://www.tcrn.unsw.edu.au/translational-research-definitions) Early translational research bridges basic/preclinical research with clinical research, looking at basic research innovations intended for proof‐of‐concept early clinical trials (gap 1). Connecting the expanding knowledge in basic cancer biology and preclinical research (involving, for instance, identification of tumour‐driving molecular pathways, new targets for treatment and biomarker research to develop predictive cancer medicine and connect to new clinical trial methodologies) requires a critical mass of expertise and resources, as well as large number of patients. To meet these demands, consortia of Comprehensive Cancer Centres (CCCs) that are ready to adopt open science and with a high profile both in basic research and early clinical trials have been established (Calvo *et al*., 2018; Forman *et al*., 2018; see also chapter byJoos *et al*., 2019).

**Figure 1:**
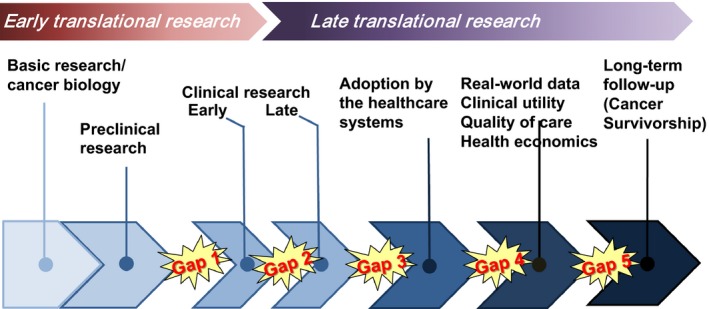
Therapeutics close up: the research continuum.

By looking closer at the cancer research continuum, it is clear that late translational research includes components that are poorly linked; in fact, there are four additional gaps (Fig. 1). The outcome of early translational research is clinical efficacy assessed on a small number of patients with limited knowledge of side effects. Late clinical research aims at demonstrating clinical effectiveness and potential added value of innovations for health care, meaning that research should deliver a ‘medical product’. Very often, this is a difficult step because it requires significant investments and almost invariably requires substantial commitments from pharma or the biotech industry (gap 2, Fig. 1). The research strategies related to late clinical research are discussed in the article by Lacombe *et al*. (2019), and the health economic aspects in the article by Jönsson and Sullivan (2019).

Innovative diagnostics and treatments should be available to patients without unnecessary delay. For several reasons, adoption of innovations by health care is the main gap that leads to substantial inequalities both within and between countries. In order to bridge a research outcome to its use by health care, the term ‘implementation research’ has been suggested (gap 3, Fig. 1). This means that implementation should follow a strict protocol regarding treatment and registration of positive and negative consequences of treatment, with the aim of assessing whether the outcome obtained in the research project(s) can be reproduced in a regular clinical care setting. The latter is an increasing problem due to the complexity of diagnostic procedures and treatment protocols when engaging in personalised/precision cancer medicine. At this stage, health economics should be taken into account. The CCC fulfil a critical role in more effectively bridging research with health care, and collaboration between CCCs will further shorten the time for innovations to reach patients.

Following a positive outcome of the implementation research, an update of the clinical guidelines addressing the new treatment should be considered (gap 4, Fig. 1). The clinical guidelines should recommend data collection for the clinical cancer registry to permit outcomes research on real‐world data. With defined and quality‐assured collection of clinical data, the clinical utility can be assessed and linked to health economics for the analysis of cost‐effectiveness and value for patients and society (see the article by Jönsson and Sullivan, 2019). With increased collaboration between CCCs, the capacity to conduct outcomes research and assess the health economic aspects of new diagnostics and treatments will be substantial, thereby providing vital information to healthcare systems for prioritisation of diagnostics and treatments.

Long‐term follow‐up of treated cancer patients is another unmet need that is discussed in the article by Lagergren *et al*. (2019) (gap 5, Fig. 1). An increasing number of patients living with a cancer diagnosis, with or without evidence of remnant disease, suffer from long‐term physical and psychosocial side effects. This is a human problem as well as a problem for healthcare systems, and more information from long‐term follow‐up of treated patients is needed to be able to draw conclusions about positive and negative effects of innovations, as well as cost‐effectiveness and value of cancer therapeutics (see article by Jönsson and Sullivan, 2019). Bridging treatment with long‐term follow‐up is considered critical, and detailed quality‐assured clinical registries have to be established to ensure that the compilation of data from centres can be readily analysed. It is suggested that this should be a task included in the accreditation methodologies for CCCs (see article by Oberst, 2019).

## 2 The cancer research continuum for cancer prevention

**Figure 2:**
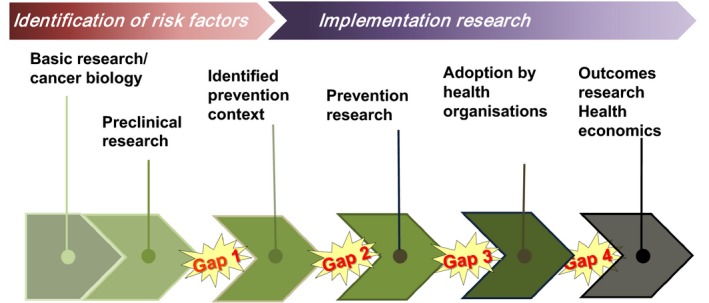
Prevention close up: the research continuum.

To increase international research collaborations and improve interaction between the different components needed for a coherent cancer prevention research continuum, Cancer Prevention Europe was created in 2018 (Forman *et al*., 2018) (see also the article by Wild *et al*., 2019 and references therein). Like in the case of therapeutics, the different components of the research continuum for cancer prevention are not sufficiently connected. Four main gaps are readily apparent between basic research and outcomes from primary, secondary and tertiary prevention strategies (Fig. 2).

Early translational research bridges basic research with a focus on the discovery of causes of cancer and the identification of risk and protective factors, with the development of a potential prevention strategy (gap 1). The new prevention strategy needs to be tested in trials aimed at assessing the efficacy (gap 2). To be adopted by health organisations, however, prevention research with documented effectiveness and potential economic consequences is required (gap 3). This gap is of great importance since knowledge from prevention research, which is estimated to be able to reduce the cancer problem by 30–40% (Forman *et al*., 2018), is currently used insufficiently, mainly as a consequence of political resistance or indifference to implement more effective measures that promote healthier lifestyles. Implemented preventive programs should be the target for outcomes research linked to health economics (gap 4).

## 3 Conclusions

There is a need to improve integration between the different components of the cancer research continuum both with regard to therapeutics and prevention. Furthermore, prevention and therapeutics have several issues in common and should work in concert. Early detection of premalignant and early invasive disease is closely connected with medical (therapeutic) prevention and secondary prevention, as well as therapeutics. For both, it will be essential to identify early disease to increase cure rates, and, in parallel, avoid overdiagnosis and overtreatment. Tertiary prevention aimed at decreasing the risk of recurrent disease is another area that will benefit from closer interactions. Outcomes research methodologies and health economics are required for demonstrating the benefits of both prevention and therapeutics. Coherent translational research both for prevention and therapeutics can help to boost multidisciplinary cancer research further and stimulate innovation. An essential prerequisite is access to optimal research environments (Ringborg *et al*., 2018; see also article by Berns, 2019).

## Conflict of interest

The author declares no conflict of interest.
